# The prospective multiple-centre randomized controlled clinical study of high-dose amoxicillin-proton pump inhibitor dual therapy for *H. pylori* infection in Sichuan areas

**DOI:** 10.1080/07853890.2022.2031269

**Published:** 2022-01-31

**Authors:** Cheng Shen, Changping Li, Muhan Lv, Xiaosong Dai, Caiping Gao, Liangping Li, Qin Zhang, Wen Pan, Chao Liu, Sijing Han, Yang Zhang, Shunbin Ding, Hong Deng, Yong Yao, Jianyu Xu, Mingyong Wei, Haiyan Shi, Peijie Yuan, Xiaoyan Yang, Yi Jian, Jing Shan, Yan Liu, Zonghua Chen, Xuejie Deng, Fei Liu, Lijuan Deng, Xianfei Zhong, Hong Li, Shaoya He, Li Chen, Gang Liu, Hairong Xu, Yuquan Zhong, Hua Shi, Jiangang Ren, The Multi-Center Clinical Research Collaboration Group of Sichuan Provincial H. pylori Scientific Group, Sichuan Provincial Medical Association

**Affiliations:** a Department of Gastroenterology, The Affiliated Hospital of Southwest Medical University, Luzhou, China; b Sichuan Provincial People's Hospital, Chengdu, China; c First People's Hospital of Liangshan Yi Autonomous Prefecture, Xichang, China; d Sichuan University West China Hospital Tibet People's Government in Chengdu Office Branch, Chengdu, China; e Chengdu Medical College Second Affiliated Hospital, Chengdu, China; f People's Hospital of Deyang City, Deyang, China; g Suining Central Hospital, Suining, China; h Sichuan Science City Hospital, Mianyang, China; i Bazhong Central Hospital, Bazhong, China; j The Fourth People's Hospital of GuangYuan, GuangYuan, China; k Chengdu Second People's Hospital, Chengdu, China; l Chengdu Third People's Hospital, Chengdu, China; m Chengdu Fifth People's Hospital, Chengdu, China; n The Second People's Hospital of Yibin, West China Hospital of Sichuan University, Yibin, China; o People's Hospital of Leshan, Leshan, China; p Leshan City Geriatric Hospital, Leshan, China; q Leshan Sichuan Armed Police General Hospital, Leshan, China; r Pengzhou People's Hospital, Chengdu, China; s Sichuan Anyue County People's Hospital, Ziyang, China; t Zigong Third People's Hospital, Zigong, China; u First Peoples Hospital of Neijiang, Neijiang, China; v The First People's Hospital of Ziyang, Ziyang, China; w Wuhou District Third People's Hospital, Chengdu, China

**Keywords:** *H. pylori*, initial treatment, amoxicillin, proton pump inhibitor, dual therapy

## Abstract

**Objectives:**

To evaluate the safety and efficacy of high-dose amoxicillin-proton pump inhibitor dual therapy, and to provide a new eradication regimen as a first-line option for patients with *H. pylori* infection.

**Methods:**

A total of 971 *H. pylori* positive patients who received initial treatment were recruited from March to August 2020, and randomly divided into treatment group and control group. The treatment group received of 20 mg esomeprazole four times daily and 750 mg amoxicillin four times daily for 14 days. Control group received of 220 mg bismuth potassium citrate twice daily, 20 mg esomeprazole twice daily, 1000 mg amoxicillin twice daily and 250 mg clarithromycin capsule twice daily for 14 days. Four weeks after the end of treatment, the urea breath test was reviewed to detect whether *H. pylori* was eradicated.

**Results:**

There were no statistical differences in age, gender, the total clinical symptom scores before and after initial treatment, the compliance, and the degree of remission of symptoms before and after initial treatment between the two groups. The eradication rates of *H. pylori* between dual therapy and quadruple therapy were 88.31% and 85.26% (*p*=.158) by intention-to-treat (ITT) analysis, 88.66% and 85.44% (*p*=.186) by modified intention-to-treat (mITT) analysis, and 91.63% and 90.60% (*p*=.116) by PP analysis, respectively. Adverse events in dual therapy group were significantly lower than quadruple therapy group (13.3% vs. 28.2% (*p*<.01)).

**Conclusions:**

For the initial treatment of *H. pylori* infection, the high-dose dual therapy regimen has the same efficacy as the bismuth-containing quadruple therapy regimen, good compliance, less adverse reactions and high safety, so it can be recommended as the empirical first-line treatment regimen for the eradication of *H. pylori* (KY2019173).

## 1. Introduction

*H. pylori* is an infectious pathogen. Eradication of *H. pylori* is not only beneficial to reduce the occurrence of *H. pylori*-related gastric diseases, prevent peptic ulcer and reduce the risk of gastric cancer [1]. It plays an important role in prevention and treatment of multi-system related diseases even for diseases outside the digestive system, such as unexplained iron deficiency anaemia of the blood system, idiopathic thrombocytopenic purpura [2], coronary atherosclerotic heart disease in circulatory system [3], and so on.

Currently, according to the Maastricht V Consensus, the Toronto Consensus and China's Fifth National Consensus Report on the treatment of *H. pylori* infection, the 14-day bismuth-containing quadruple therapy is recommended as the first choice for empirical treatment [4–6]. However, with the continuous increase of the resistance rate of *H. pylori* to clarithromycin, levofloxacin, metronidazole and other antibiotics, the eradication rate gradually decreased [6,7]. Bismuth-containing quadruple therapy regimen has some limitations: variety of drugs, complex regimens, many drug-related side effects, poor compliance, high antibiotic resistance rate and high total cost of drug. If the treatment fails, it will also increase the secondary resistance to antibiotics, resulting in obvious limitations of subsequent remedial eradication options. This prompted us to explore new treatments for the eradication of *H. pylori*.

At present, the primary drug resistance rate of amoxicillin to *H. pylori* in China is very low (0–5%) [8], and amoxicillin has a strong anti-*H. pylori* effect, is not easy to produce drug resistance, and the incidence of adverse reactions is small, so it is the preferred antibiotic for the eradication of *H. pylori* treatment. As early as 1989, the dual therapy regimen containing amoxicillin and PPI was used to eradicate *H. pylori*, but due to the low eradication rate, only 54.8–61.9%, and then gradually replaced by triple (PPI + two antibiotics) and quadruple regimen (bismuth + PPI + two antibiotics) [9,10]. In recent years, with the change of the situation of drug resistance in *H. pylori*, dual therapy had been proposed again and improved, but its efficacy is controversial. Some research has shown that for initial treated patients with *H. pylori* eradication, the dual therapy with high doses of PPI and amoxicillin has similar or even higher eradication rates than the classical bismuth-containing quadruple regimen [11–15], but based on small sample size and most of them are single-centre studies. There are also great differences in efficacy, so the purpose of this study is to conduct multicentre randomized controlled studies, expand the sample size, and evaluate the efficacy and safety of high-dose dual therapy as the first choice for patients with *H. pylori*, and whether it is superior to the current experience of first-line eradication of *H. pylori* with bismuth-containing quadruple regimen, so as to draw the conclusion that high-dose dual therapy can become the first choice of the new *H. pylori* eradication regimen.

## 2. Materials and methods

### 2.1. Study design and ethics

This study was a prospective, open-label, multicentre, randomized controlled trial, which was approved by the Clinical Trials Ethics Committee of the Affiliated Hospital of Southwest Medical University on 17 December 2019. All subjects signed their written informed consent before enrolment.

### 2.2. Subjects

A total of 971 *H. pylori* positive patients who received initial treatment at 23 Grade 3A hospitals in Sichuan Province, China, were recruited in this study from March to August 2020. Patients were considered eligible for enrolment if they satisfies all of these conditions: (1) age range of 18–70 years; (2) take no medication which including antibiotic, bismuth and traditional Chinese medicine before 4 weeks of treatment; (3) taken no medication which effect on *H. pylori* activity, such as proton pump inhibitors (PPIs), H2-receptor antagonist, etc. before 2 weeks of treatment; (4) patients who have not received previous eradication treatment for *H. pylori* can be diagnosed as *H. pylori* positive with one of the following positive results: 13C/14C-urea breath test (UBT), rapid urease test (RUT) and histological analysis. Patients were excluded if they satisfies any of the following: (1) severe cardiac, pulmonary and renal insufficiency, immunocompromised; (2) drug allergy contraindications; (3) mental illness and communication disorders; (4) pregnancy and lactation; (5) complicated with organic lesions such as digestive tract tumour and gastrointestinal bleeding. Quit-out criteria: (1) patients automatically asked to withdraw from the study; (2) patients had unknown adverse events.

### 2.3. Randomization and intervention

The eligible subjects were randomly divided into two groups according to the ratio of 1:1 by a computer-generated randomized digital table. The treatment group (dual therapy) received of 20 mg esomeprazole AstraZeneca Pharmaceutical Co., Ltd., Cambridge, UK, www.astrazeneca.com.cn) four times daily and 750 mg amoxicillin (Kunming Baker Norton Pharmaceutical Co., Ltd., Kunming, China, www.kbn.com.cn) four times daily for 14 days. Control group (bismuth-containing quadruple therapy) received of 220 mg bismuth potassium citrate (Lizhu Group Lizhu Pharmaceutical Factory, Guangzhou, China, www.livzon.com.cn) twice daily, 20 mg esomeprazole (AstraZeneca Pharmaceutical Co., Ltd., Cambridge, UK, www.astrazeneca.com.cn) twice daily, 1000 mg amoxicillin (Kunming Baker Norton Pharmaceutical Co., Ltd., Kunming, China, www.kbn.com.cn) twice daily and 250 mg clarithromycin capsule (Shanghai Abbott Pharmaceutical Co., Ltd., Shanghai, China, www.abbott.com.cn) twice daily for 14 days. Bismuth potassium citrate and esomeprazole were taken half an hour before meals, while amoxicillin and clarithromycin were taken half an hour after meals.

### 2.4. Follow-up

During treatment, all subjects need to be followed up through Wechat, telephone, etc., to record the use of drugs and adverse reactions. Patients should be reminded to strictly limit smoking and alcohol during medication and within 1 week after drug withdrawal. At least 4 weeks after treatment, patients should come to the hospital again to review the eradication of *H. pylori*.

### 2.5. Outcomes

The primary outcome measure in this study was the *H. pylori* eradication rate between high-dose dual therapy group and bismuth-containing quadruple therapy group. Four weeks after treatment, the negative results of 14C-UBT or 13C-UBT were judged as successful eradication and positive as failed eradication.

The secondary outcome measures were the degree of symptoms relief, compliance and adverse events. The therapeutic effect observation table (Table 1) was used to evaluate the symptom scores of all subjects before and after treatment, including epigastric pain, postprandial discomfort, loss of appetite, etc., in which 0 point: no symptoms; 1 point: mild symptoms; 2 points: moderate, tolerable; 3 points: serious, affecting work and life; 4 points: severe, unable to live normal life; the total symptom score is the sum of all symptom scores. The total symptom score of the patients is 1/3 of the pre-treatment score and less than that of the pre-treatment score for markedly effective. The score after treatment is 2/3 or less than that before treatment for effective; the score after treatment is more than 2/3 of that before treatment for ineffective. Take the markedly effective and effective as the clinical total effective. The compliance was measured by the medication use rate during the treatment period of the subjects, that is, the ratio of the actual amount of medication taken by the patient to the total amount of medication to be taken during the 14-day treatment period, was measured by counting the number of tablets. On evaluating and recording adverse effects, according to the effects of adverse reactions on daily life, they were divided into "mild (transient, tolerable and does not affect daily life)", "moderate (psychological or physical discomfort, partly affect daily life)” and "severe (severe discomfort, daily life cannot be carried out normally)”.

**Table 1.** Therapeutic effect observation table.

Symptoms	Pre-treatment score	Post-treatment score	Curative
Nausea			
Vomiting			
Loss of appetite			
Abdominal pain			
Abdominal distension			
Diarrhoea			
Constipation			
Belching			
Acid regurgitation			
Bitter taste			
Heartburn			
Total score			

Evaluate the symptom scores of all subjects before and after treatment. 0 point: no symptoms; 1 point: mild symptoms; 2 points: moderate, tolerable; 3 points: serious, affecting work and life; 4 points: severe, unable to live a normal life. The total symptom score is the sum of all symptom scores. Markedly effective: the total symptom score of the patients is 1/3 of the pre-treatment score and less than that of the pre-treatment score. Effective: the score after treatment is 2/3 or less than that before treatment. Ineffective: the score after treatment is more than 2/3 of that before treatment. Take the markedly effective and effective as the clinical total effective.

### 2.6. Statistical analysis

All statistical analyses were performed using SPSS22.0 statistical software (SPSS Inc., Chicago, IL), the eradication rate of *H. pylori* was analysed by intention-to-treat (ITT) analysis of intended treatment (including all subjects who met the criteria), modified intention-to-treat (mITT) analysis (analysis of subjects receiving treatment) and per-protocol (PP) analysis (analysis of subjects who received treatment and re-examined 13C-UBT or 14C-UBT 4 weeks after the end of treatment. Excluding people who dropped out, lost follow-up and not re-examined). For the comparison of the differences between groups, the counting variables obey the normal distribution by *t*-test, and the results are described by mean ± standard deviation, while the non-normal distribution data are described by rank sum test, and the results are described by median and quartile spacing. Chi-square test or Fisher's exact test was used to classify variables, and the results were described by percentage or frequency. Bilateral *p*<.05 considered that the difference was statistically significant.

## 3. Results

### 3.1. Baseline characteristics of the subjects

**Figure 1.**
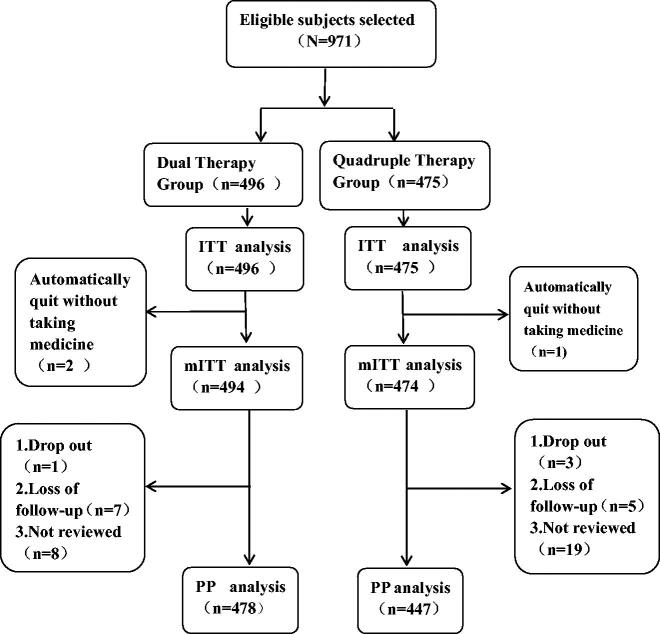
Analysis flowchart. ITT: intention-to-treat; mITT: modified intention-to-treat; PP: per-protocol.

A total of 971 subjects were enrolled in this study, including 496 high-dose dual therapy and 475 bismuth-containing quadruple therapy. A total of three people quit automatically without taking medicine, including two in dual therapy and one in bismuth-containing quadruple therapy. Four people dropped out (two people quit due to serious adverse reactions in the quadruple group and one person in the dual group; one person in the quadruple group quit due to interruption of medication), 12 lost follow-up and 27 were not re-examined (the research flowchart is shown in Figure 1). There was no statistical difference in the distribution of age, sex and the total score of clinical symptoms before and after initial treatment between the two groups (shown in Table 2).

**Table 2.** Demographic characteristics and symptom score.

General characteristics	Dual therapy group	Quadruple therapy group	*p* Value
Gender			.689
Male	212	197	
Female	284	278	
Age			.640
Mean ± SD	46.65 ± 13.840	46.23 ± 14.202	
Total score of symptoms before initial treatment (median and quartile spacing P25–P75)	5 (2–8)	4 (2–8)	.890
Total score of symptoms after initial treatment (median and quartile spacing P25–P75)	1 (0–2)	1 (0–2)	.091
The degree of symptoms relief			.451
Markedly effective	57.9%	53.3%	
Effective	18.5%	19.8%	
Ineffective	2.8%	4%	
Not marked	20.8%	22.9%	
Adverse events			<.01
Yes	13.3%	28.2%	
No	86.7%	71.8%	
Compliance			.951
Good	99.19% (492/496)	99.16% (471/475)	
Bad	0.81% (4/496)	0.84% (4/475)	

### 3.2 *H. pylori* eradication rates

In ITT analysis, the eradication rate of *H. pylori* was 88.31% in the dual therapy group and 85.26% in the bismuth-containing quadruple therapy group (*p*=.158). In mITT analysis, the eradication rate of *H. pylori* was 88.66% in the dual therapy group and 85.44% in the bismuth-containing quadruple therapy group (*p*=.186). In the analysis of PP (including one patient who was successfully eradicated but was excluded from the experiment due to adverse disease), the eradication rate of *H. pylori* was 91.63% in the dual therapy group and 90.60% in the bismuth-containing quadruple therapy group (*p*=.116). The analysis of the three schemes showed that there was no statistical difference in the success rate of *H. pylori* eradication between the two groups (the analysis of *H. pylori* eradication rate in the two groups is shown in Table 3).

**Table 3.** Eradication rate of *H. pylori*.

Analysis method	Dual therapy group	Quadruple therapy group	*p* Value
ITT	88.31% (438/496) (85.6–93.2%)	85.26% (405/475) (84.1–91.7%)	.158
mITT	88.66% (438/494) (86.3–93.8%)	85.44% (405/474) (84.5–91.9%)	.186
PP	91.63% (438/478) (87.4–95.1%)	90.60% (405/447) (86.6–94.2%)	.116

### 3.3. The degree of symptoms relief

After two different regimens for eradication of *H. pylori*, the degree of remission of clinical symptoms before and after treatment (shown in Table 2). The effects of the dual and quadruple groups in different degrees are as follows: DT vs. QT: markedly effective: 57.9% vs. 53.3%; effective 18.5% vs. 19.8%; ineffective: 2.8% vs. 4%; not marked: 20.8% vs. 22.9% (*p*=.451). The difference is not statistically significant.

### 3.4. Compliance and adverse events

About compliance, there were three patients who did not start medication and led to automatic withdrawal, two in the dual group and one in the quadruple group. Irregular medication: one patient in the dual group and one patient in the quadruple group. In the process of eradication, a total of three people dropped out of the study due to serious adverse reactions, including one in the dual group and two in the quadruple group. To sum up, the compliance of dual group and quadruple group was 99.19% and 99.16%, respectively (*p*=.951). Both groups had good compliance, and the difference was no statistically significant. The incidence of adverse events in the dual group and the quadruple group was 13.3% and 28.2%, respectively (*p*<.01), and the difference was statistically significant. Common symptoms of ADR are: bitter taste (51/971), diarrhoea (36/971), abdominal distension (29/971), black stool (26/971), dry mouth (21/971), nausea (17/971), abdominal pain (13/971), loss of appetite (12/971), belching (11/971), bad breath (9/971), vomiting (8/971), acid regurgitation (8/971), constipation (7/971), insomnia (7/971), fatigue (6/971), dizziness (5/971), headache (4/971), blackened tongue coating (3/971), swallowing discomfort (2/971), yellow urination (2/971), etc. The rare symptoms were chest tightness (1/971), taste change (1/971), heartburn (1/971), skin pruritus (1/971), paresthaesia of limbs (1/971), drowsiness (1/971), etc. There were statistically significant differences in the occurrence of ADR symptoms between the two groups, such as bitter taste (4/496 vs. 47/475, *p*<.01), abdominal pain (3/496 vs. 10/475, *p*=.042), belching (2/496 vs. 9/475, *p*=.028), black stool (4/496 vs. 22/475, *p*<.01) and loss of appetite (2/496 vs. 10/475, *p*=.016), others were no statistical difference (shown in Table 4). After drug withdrawal, the symptoms of ADR of all patients disappeared, and no serious adverse events occurred.

**Table 4.** Adverse events.

Adverse events	Dual therapy group	Quadruple therapy group	*p* Value
Diarrhoea	15	21	.249
Abdominal distension	13	16	.494
Abdominal pain	3	10	.042
Dizziness	2	3	.619
Bitter taste	4	47	<.01
Nausea	5	12	.071
Headache	3	1	.338
Constipation	4	3	.747
Belching	2	9	.028
Swallowing discomfort	1	1	.976
Vomiting	3	5	.440
Acid regurgitation	4	4	.951
Chest tightness	1	0	.328
Heartburn	0	1	.307
Bad breath	3	6	.285
Insomnia	5	2	.280
Fatigue	3	3	.958
Black stool	4	22	<.01
Paresthaesia of limbs	1	0	.328
Drowsiness	0	1	.307
Skin pruritus	1	0	.328
Dry mouth	9	12	.446
Loss of appetite	2	10	.016
Taste change	0	1	.307
Blackened tongue coating	0	3	.076
Yellow urination	1	1	.974

## 4. Discussion

This is a multicentre randomized controlled clinical trial comparing the efficacy and safety of the high-dose amoxicillin combined with esomeprazole for *H. pylori* eradication versus the classic bismuth-containing quadruple regimen of bismuth potassium citrate combined with esomeprazole combined with amoxicillin combined with clarithromycin in patients with *H. pylori* infection who are first treated. In this study, we can see: (1) in the eradication rate of *H. pylori*, high-dose dual therapy and bismuth-containing quadruple therapy through ITT, mITT, PP analysis, show that there is no statistical difference between them, and the efficacy was similar. (2) In terms of the degree of symptom relief: both high-dose dual therapy and bismuth-containing quadruple therapy can effectively alleviate the persistent state of symptoms before treatment and greatly reduce the total score of individual symptom severity, and there is no significant difference between them. (3) In terms of compliance: the two regimens have good compliance, and the difference is not statistically significant. However, high-dose dual therapy has fewer types of drugs, relatively simple drug regimen, easy to remember and implement, and relatively low drug cost, which may be the preferred choice for more people. (4) In terms of adverse events: due to the relatively more types of drugs used in the bismuth-containing quadruple regimen, the adverse reactions were also increased. Compared with high-dose dual therapy, it led to more adverse reactions, there was a significant difference in the incidence of adverse events between the two groups, especially in bitterness, abdominal pain, belching, black stool and loss of appetite. Relatively speaking, high-dose dual therapy has higher safety. In summary, high-dose dual therapy can become a first-line treatment for the eradication of *H. pylori*.

High-dose dual therapy is a modified regimen designed to improve the eradication rate of *H. pylori* based on the basic properties of the two drugs. Amoxicillin is a pH-dependent and time-dependent antibiotic [16]. It has anti-bacterial effect only when its blood concentration is maintained above the minimum inhibitory concentration (MIC). Strong bactericidal effect can be obtained when the blood concentration of amoxicillin is 10 times higher than the MIC, since it is generally rapidly absorbed and excreted within approximately 6–8 h after administration. In order to increase and maintain blood concentration, we chose to administer it four times a day (6–8 h apart) at a single dose of 750 mg instead of twice a day. In addition, only in the gastric environment with pH >6 can amoxicillin have stable antibiotic activity [16]. While the stomach is a strong acid environment, which is necessary for the rational application of combined PPI [16–18], PPI inhibits the secretion of basal gastric acid and irritant gastric acid by covalently binding with H^+^/K^+^ ATP-ase in gastric parietal cells. Its acid inhibition effect is strong and lasting for a long time [19], which depends on the drug concentration of PPI, which is related to the dose dependence of PPI and is also affected by CYP2C19 gene polymorphism. CYP2C19 participates in the metabolism of PPI, and its genetic polymorphism determines the activity of metabolic enzymes. Studies have shown that repeated administration and enhanced dose administration can make the blood concentration and acid inhibition of PPI in the stomach least affected by CYP2C19 genotyping status [20,21]. There is even a randomized controlled trial in Japan showing that esomeprazole is administered 20 mg each time, four times a day. Strong acid inhibition could be achieved in the stomach of all subjects without being affected by CYP2C19 gene polymorphism [22]. Therefore, in this study, we selected PPI: esomeprazole, which is less affected by CYP2C19 gene polymorphism, and administered 20 mg, four times a day to increase gastric pH, to make gastric pH >6, increase the absorption of amoxicillin, but also improve its own anti-*H. pylori* effect.

The determination of the 14-day treatment time is recommended in the current guidelines [4] unless 10-day local treatment is proved to be effective. Similarly, for the high-dose dual therapy, Zullo et al. [23] confirmed that 10-day high-dose dual therapy with esomeprazole and amoxicillin can achieve a high eradication rate (close to 90%), and suggested that a longer 14-day regimen may be more effective, especially in individuals with high drug tolerance. Zhang et al. [24] also compared the 10-day QID dual therapy and 14-day TID dual therapy, and concluded that although there was no significant difference in the eradication rate between the two groups, the eradication rate of 10-day treatment group was lower than 14-day treatment group, and failed to achieve an acceptable efficacy, the eradication rate was less than 80%. This may be related to the re-growth of some dormant bacteria that cannot be eradicated. Therefore, the choice of treatment time of 14 days is appropriate.

In recent years, the decrease in the eradication rate of *H. pylori* is mainly caused by the increase of antibiotic resistance on one hand, and the poor compliance due to the numerous adverse events occurring in the eradication regimen on the other hand [13]. One advantage of high-dose dual therapy is that it chooses amoxicillin, an antibiotic with low primary and secondary resistance rates in China. Thus, the application of clarithromycin, metronidazole and levofloxacin that have produced high drug resistance rates is reduced, the unreasonable application of antibiotics is reduced, and the growth trend of drug resistance is slowed down. After the failure of initial treatment, there are more remedial options, and drug sensitivity tests can also be conducted to improve the eradication rate [6]. Another prominent advantage is the significant reduction in the incidence of adverse events. We can see two regimens have good compliance (99.19% vs. 99.16%), such good compliance may be related to the regular medication guidance and follow-up for all subjects by Wechat and telephone during medication. At the same time, we also can clearly see the application of bismuth-containing quadruple therapy has dramatically increased the incidence of adverse events (about 28.2%), with bitter taste, abdominal pain, belching, black stool, loss of appetite common and significant. It has a variety of medications and complex regimen, this will not only reduce compliance, but also increase the requirements for the application of the regimen, for those elderly patients with more basic diseases and liver and kidney insufficiency, its application may be limited. While the high-dose dual therapy regimen has fewer drugs and is simple, excluding people who are allergic to penicillin drugs, basically all patients can be used, which is conducive to promotion.

At present, the evaluation of the efficacy of high-dose dual therapy to eradicate *H. pylori* is a research hotspot. Numerous studies at home and abroad have different drug regimens, types and courses of PPI, and the results obtained are also uneven. Now, all studies on high-dose dual therapy in China are single-centre studies. For example, a 14-day clinical control study of dual therapy (esomeprazole and amoxicillin four times a day) by Song et al. shows that the eradication rate of *H. pylori* can reach 87.1% [13]; Yang et al. compared the efficacy and safety of dual therapy, and showed that the eradication rate of *H. pylori* could be 87.9% after 14 days of treatment, and 79.8% after 10 days of treatment [14,24]. Tai et al. even achieved 91.7% *H. pylori* eradication rate through the comparison of modified 14-day dual therapy (esomeprazole 40 mg, three times a day, amoxicillin 750 mg, four times a day) [25]; Yang et al. [11], Ren et al. [26] and others also achieved high eradication rate by using high-dose of rabeprazole combined with amoxicillin. Although these studies have achieved acceptable eradication rate of *H. pylori*, they are all single-centre studies, which lack universality, and need larger sample size and multi-centre clinical trials to further verify. Recently, Suzuki et al., in a multicentre study in Japan, showed that in areas with high clarithromycin resistance, the seven-day vonoprazan and low-dose amoxicillin dual therapy provided acceptable *H. pylori* eradication rates (87.1%) [27]. The conclusions drawn from the above studies are consistent with the present study to a certain extent, that is, the high-dose dual therapy has similar or even higher eradication rate of *H. pylori* compared with the currently recommended first-line therapy, without significant difference. Compared to them, our high-dose dual therapy of *H. pylori* eradication rate is similar. China is a country with high clarithromycin resistance (approximately 20–45%), and there are regional differences. There are many reports on drug resistance of *H. pylori*, but most of them come from economically developed areas, while there are few reports about Sichuan Province in southwest China. A study [28] showed that *H. pylori* in southern Sichuan province had the metronidazole resistance rate of 86.25%, followed by levofloxacin 67.5%, clarithromycin 28.75%, tetracycline 7.50% and amoxicillin 5.00%. Except that the resistance rate of levofloxacin was significantly higher than that of the domestic overall level, the resistance rate of the other four antibiotics was similar to that of the domestic overall level. Sichuan is a region with high clarithromycin resistance (>15%), but due to the limitations of drug sensitivity test in China, the bismuth-containing quadruple regimen is the first choice for empirical therapy in the absence of drug sensitivity results. Compared with the resistance rate of other antibiotics, the resistance rate of clarithromycin is relatively low and easy to obtain, so the quadruple regimen containing clarithromycin was selected as the efficacy control. This study shows that the optimized dual regimen can achieve the same or even higher eradication rate as the bismuth-containing quadruple regimen, which is undoubtedly a new treatment choice. This clinical trial of high-dose dual therapy is the first multicentre study in China, and there is no unified standard about the medication scheme of dual therapy; we need more and larger-scale experiments to explore and clarify, which is of great significance to the follow-up related research.

There are some limitations in this study. Most of our subjects identified *H. pylori* infection by 13C or 14C-UBT, most of them had no pathological diagnosis, and did not carry out *H. pylori* culture and identification and related drug sensitivity tests. On the one hand, UBT is the most commonly used non-invasive test to detect *H. pylori* in clinical. The operation is simple, the accuracy is high and is not affected by the focal distribution of *H. pylori* in the stomach, but when the detection value is close to the critical value, the reliability is poor [29]. On the other hand, according to the fifth national consensus report on the treatment of *H. pylori* infection in China, for areas with high clarithromycin resistance rate (>15%), individual drug sensitivity tests should be carried out before treatment, otherwise the *H. pylori* eradication rate of the classic bismuth-containing quadruple regimen will be greatly reduced and the results will be affected. However, due to the low availability and accuracy of drug sensitivity tests, the cost–benefit ratio is still controversial. It is difficult to be widely used in initial treated patients [6]. Clinically, antibiotic susceptibility tests are not routinely carried out, but Sichuan is a region with high resistance to clarithromycin, makes it difficult for us to evaluate the effect of clarithromycin resistant strains on the eradication rate of *H. pylori*. In addition, amoxicillin and PPI reached sufficient blood concentration in high-dose dual therapy, which is an important condition of the regimen works. CYP2C19 genotyping may be an influencing factor of the study. All the subjects in this study are Chinese population, and most of their CYP2C19 genotypes are slow metabolic type and intermediate metabolic type, due to the limitation of experimental conditions, we did not monitor the genotyping of CYP2C19 and gastric pH and two drugs of blood drug concentration. For people with fast and ultrafast metabolic CYP2C19 genes, the drug concentration of PPI in the stomach may be difficult to reach or maintain the environment where pH >6. Although we have used PPI, which is less affected by the status of CYP2C19 genotyping in this study, this difference still exists. There have been many studies [13,30] to apply rabeprazole (mainly through non-enzymatic pathway metabolism, less affected by CYP2C19 gene) on the basis of combination of amoxicillin dual therapy that has also achieved good results. At the same time, there has been a new potassium competitive acid blocker, vonoprazan, which is mainly metabolized by CYP3A4 pathway into inactive metabolites, little affected by CYP2C19 gene polymorphism and can be ignored. Vonoprazan has a stronger and more lasting effect on inhibiting gastric acid compared with PPI [31]. However, due to the high price and serious adverse reactions, the use of this kind of drug in China is still controversial. It may be able to reduce the impact of CYP2C19 genotyping differences and optimize the dual therapy again, but this still needs more experiments to explore. As far as this study is concerned, due to the unknown of these factors, we cannot know whether the effect of the drug is consistent with the experimental results, which needs to be further improved.

## 5. Conclusions

In conclusion, for the initial treatment of *H. pylori* infection in Sichuan, China, the high-dose dual regimen and the bismuth-containing quadruple regimen have similar efficacy, good compliance, fewer adverse events and higher safety, so they can be recommended as the empirical first-line regimen for the eradication of *H. pylori* infection.

## Data Availability

The data that support the findings of this study are available on request from the corresponding author. The data are not publicly available due to privacy or ethical restrictions.
